# Physiological Impairment and Biochemical Modifications Induced by Triclosan in Mediterranean Mussels

**DOI:** 10.3390/ani13040583

**Published:** 2023-02-07

**Authors:** Imen Bouzidi, Karine Mougin, Hamouda Beyrem, Mohammed I. Alghonaim, Sulaiman A. Alsalamah, Ashraf A. Qurtam, Ezzeddine Mahmoudi, Fehmi Boufahja, Badreddine Sellami

**Affiliations:** 1 Laboratory of Environment Biomonitoring, Coastal Ecology Unit, Faculty of Sciences of Bizerta, University of Carthage, Zarzouna 7021, Tunisia; 2 Institut Supérieur de Biotechnologies de Béja, Université de Jendouba, Jendouba 8189, Tunisia; 3 Institut de Science des Matériaux, Université de Haute Alsace, IS2M-CNRS-UMR 7361, 15 Rue Jean Starcky, 68057 Mulhouse, France; 4 Biology Department, College of Science, Imam Mohammad Ibn Saud Islamic University (IMSIU), Riyadh 11623, Saudi Arabia; 5 Institut National des Sciences et Technologies de la Mer, Tabarka 8110, Tunisia

**Keywords:** pharmaceuticals, *Mytilus galloprovincialis*, biomarkers, biomonitoring

## Abstract

**Simple Summary:**

The use of physiological and biochemical biomarkers in studying the effects of endocrine disruptors on aquatic invertebrates have rarely been adopted by scientists despite their obvious advantage. Applying such tools is discernible in reducing health risks for humans commonly consuming seafood such as bivalves and crustaceans. The outcomes of the current work support the use of the Mediterranean mussels as bioindicators of the presence of triclosan in the environment, and validate the usefulness of biomarkers and physiological features in determining thresholds and government policies.

**Abstract:**

The effects of pharmaceutical under aquatic biota are still not well established. In this investigation, we assessed the results of a common pharmaceutical’s, triclosan (TCS), treatment on physiological and biochemical status of the Mediterranean mussels. Filtration and respiration rates were statistically reduced after treatment with highest considered concentration TCS2 = 100 µg·L^−1^. However, no modification (*p* > 0.05) was detected after treatment with TCS1 = 50 µg·L^−1^. For biochemical responses, oxidative stress parameters including H_2_O_2_ level and antioxidant enzymes were enhanced following concentration in considered organs. In parallel, Malondialdheyde content was measured in mussels after TCS treatment and lipid peroxidation occurred at high TCS concentration. Neurotoxicity evaluated by acetylcholinesterase (AChE) activity was induced in gills and digestive glands after exposure to TCS2. Overall, physiological impairment, oxidative stress, lipid peroxidation and neurotoxicity could be induced by triclosan in mussels. The association of physiological and biochemical biomarkers constitute a useful tool to measure the impact of pharmaceuticals in marine organism.

## 1. Introduction

Various therapeutic and diagnostic uses of pharmaceuticals lead to the discharge of these products which eventually reach the marine environment. The origin of pharmaceuticals contamination of marine ecosystems is mainly continental, carried by rivers but also by wastewater from coastal cities, treated or not, discharged directly at sea through outfalls.

These chemicals can have profound biological effects [1], causing additional stress on marine life already affected by other sources. In addition, it is anticipated that as the population grows, the effects of pharmaceuticals on coastal habitats would worsen [2]. Furthermore, it is impossible to ignore the mounting pressure from human activities, which have important ramifications for assessing ocean risk and recognizing potential negative effects on the environment and on human health.

Among pharmaceuticals, antimicrobial agent contamination is a recent problem affecting both public health and the environment [3]. These substances are already largely used in consumer goods [4]. Due to their widespread distribution, they have been recorded in aquatic environments [5].

Triclosan (TCS) is an antimicrobial compound utilized in a number of home items, as well as in plastic products including toys for children and kitchenware [4]. This drug was used since the 1970s, and heavily in hundreds of everyday chemicals since the 1990s. This antimicrobial agent is detectable in waterways [6]. TCS is currently one of the compounds that has attracted the attention of several environmental agencies [7]. It has been identified in different aquatic compartments in very important amount [8,9]. The concentration of this antimicrobial in aquatic system varies from nanogram to microgram [10].

TCS is a hormone disruptor necessary for healthy growth and reproduction [11]. It penetrates the aquatic system through a variety of channels and poses a serious threat to the marine organism [6]. It adsorbs to soil and sediment and can become bioaccumulative [12]. Researchers are more interested in the existence of triclosan and its potential consequences [13,14]. The sublethal effects of antimicrobial agents on the physiological or metabolic parameters of aquatic organisms are essential for the evaluation of their chemical toxicity. These effects often occur at low concentrations and may be linked to the health of individuals and therefore to the health of the population. Physiological responses and oxidative biomarkers are pertinent parameters to identify environmentally stressful conditions [15,16,17,18,19].

Bivalves could be used to measure environmental chemical exposure [15]. These sentinel species are crucial biomonitoring tools because they absorb contaminants from the environment in their tissues [15]. Depending on how quickly a drug is absorbed, mussels may be more or less susceptible to its negative physiological and molecular effects.

The Mediterranean mussel (*Mytilus galloprovincialis*) is a good bioindicator species because of its broad distribution, long lifespan, and capacity to filter out pollutants from marine water [16]. This species is one of the most popular marine organisms used for environmental pollution quantification [17]. Additionally, given that this species is consumed globally, pharmaceutical contamination may endanger the environment and humankind.

The lack of knowledge regarding the consequences of TCS on marine organisms, especially on the physiological and metabolic parameters of aquatic organisms, makes the need for the availability of ecotoxicological data for this antimicrobial agent to be expanded.

In the current study, changes in the physiology and biochemical state after TCS contamination were examined using filtration and respiration rates and the oxidative biomarker analysis to underline the harmful effects of TCS in bivalves. This investigation could provide information on possible risks that could arise to marine organisms when exposed to this chemical.

## 2. Material and Methods

### 2.1. Specimens’ Collection and Exposure Preparation

Mussels *Mytilus galloprovincialis* of similar size (shell length 45–55 mm) were collected from Bizerte lagoon, Tunisia (37°13′19.26″ N 9°55′46.24″ E). Upon return to the laboratory, the external side of the bivalves was polished. They were thereafter distributed in glass tanks (3 Liter volume) for an acclimatization under environmental condition for a week prior to TCS contamination. During the experiment, mussels lived in natural seawater which was renewed every two days.

Due to the fact that dimethyl-sulfoxide (DMSO) had no discernible impact on the biomarker responses, stock TCS solutions (purity 98%) were prepared by first dissolving TCS in DMSO, and then added to tanks [20]. Additionally, it has been demonstrated that the marine bivalves’ metabolic profiles were unaffected by low concentration of DMSO [21]. DMSO and TCS (purity ≥ 96%) were purchased from Sigma-Aldrich, Co., St. Louis, MO, USA.

Firstly, we evaluated the physiological impact of TCS measuring the filtration and respirations capacities of mussels. To this aim, mussels (three replicates of 10 individuals per condition) were treated during 14 days to 0, 0 + DMSO, TCS1 = 50 µg·L^−1^ and TCS2 = 100 µg·L^−1^. The TCS concentrations were obtained after dissolution of 20 mg of TCS in 1 mL of DMSO. Exposures were performed under semi-static conditions, with daily changes of the entire volume of water and the addition of TCS stock solution in order to yield the final test nominal concentrations. A DMSO-treated group (15 µL/3 L = 5 µg·L^−1^) was established to examine the effects of the solvent and this represents the highest volume of DMSO used in the present study to allow a concentration 100 µg·L^−1^ TCS/seawater (TCS2). In parallel, to measure oxidative stress, lipid peroxidation, and neurotoxicity induced by TCS, three replicates of 10 individuals per condition were considered. During the experimental period, salinity, temperature, dissolved oxygen, and pH were measured daily with a thermo-salinity meter (LF196; WTW, Weilheim, Germany), an oximeter (OXI 330/SET, WTW), and a pH meter (pH 330/SET-1, WTW), respectively. The temperature was maintained at 19 ± 2 °C, oxygen at 6.2 mg/L, and the salinity was 32‰. After exposure to TCS, tissues were excised, gills and digestive glands were then stored at −80 °C.

### 2.2. Physiological Parameters Determination

The physiological effects of TCS on mussels were determined by measuring the rates of respiration and filtration. Filtration rate measurements were made in closed chambers on the basis of the removal of neutral red dye (CAS: 553-24-2 purchased from Sigma-Aldrich) from the water column as reported in Coughlan [22]. Following exposure, 30 mussels from each treatment were individually distributed in 200 mL beakers that contained 100 mL of neutral red solution (1 mg·L^−1^). An aliquot of water was taken out of each beaker before adding the mussels to the solution in order to figure out the initial concentration (C0). The bivalves were taken out after 2 h; the residual solution (Ct) and initial aliquot (C0) were then acidified to pH 5 with 5% HCl. In order to determine the amounts of neutral red, the absorbance was finally measured at 550 nm. Standards of neutral red were measured along with the samples and used to establish a standard curve, from which dye concentrations could be extrapolated. FR was calculated using the following equation:

FR = [M/nt] log (C0/Ct)(1)

where FR is the filtration rate (mg·indiv^−1^·h^−1^), M is the total volume of water, n is the number of clams used, t is the time in h and C0 and Ct the concentration values between two sampling times.

The respiration rate was measured as reported by Basti et al. [23], using calibrated oxygen electrodes linked to an oximeter. The reduction in oxygen concentrations was measured every half hour for three hours and calculated using the following equation:

RR = [Ct0 − Cti] × V/(ti − t0)(2)

where RR is the respiration rate (mg·O_2_·h^−1^), Ct is the concentration of oxygen in the water (mg·O_2_·L^−1^) at time t (t0: initial time and ti: end time h) and V is the volume of the total solution in the sealed chamber.

### 2.3. Biomarker’s Determination

Digestive glands and gills of 30 fresh bivalves were separately homogenized into 100 mM phosphate buffer (pH 7.0) and centrifuged at 10,000 g for half an hour at 4 °C. The supernatants obtained were considered to measure H_2_O_2_ level, the activities of superoxide dismutase (SOD), glutathione-S-transferase (GST), catalase (CAT), and acetylcholinesterase (AChE), and the level of malondialdehyde (MDA).

The content of H_2_O_2_ was determined according to the Wolff method [24]. SOD activity was measured by the pyrogallol method in U·mg^−1^ protein as described Marklund and Marklund [25]. CAT activity was evaluated in nMol·min^−1^·mg^−1^ protein according to the Aebi method [26]. The reaction solution was composed of 80 mM phosphate buffer, pH 6.5, and 50 mM H_2_O_2_ [27]. Following the GSH’s conjugation with 1-chloro-2,4-dinitrobenzene, the GST activity was measured at 340 nm [28]. AChE activity was evaluated according to the Ellman et al. [29] method and given in nMol·min^−1^·mg^−1^ protein. The intensity of lipid peroxidation was estimated according to Tkachenko and Grudniewska [30] and mean values were reported as nmol MDA per mg of protein. The content of proteins was measured by using as standard bovine serum albumin (BSA) as described by Bradford [31].

All reagents and equipment needed for biochemical determination were purchased from Sigma-Aldrich, Co., St. Louis, MO, USA.

### 2.4. Statistical Analyses

STATISTICA 8.0 software was used to conduct the statistical difference between experimental conditions. A one-way ANOVA was employed and Tukey’s HSD test was considered to find out which average values differed discernibly. The significance threshold was set at *p* < 0.05 for all statistical analyses.

## 3. Results

### 3.1. Triclosan Impact on Mussel’s Physiology

**Figure 1:**
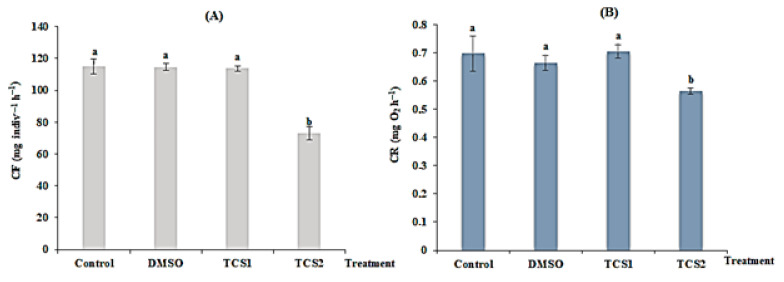
Filtration rate (FR, (**A**)) and respiration rate (RR, (**B**)) of untreated mussels and mussels treated with DMSO (5 µg·L^−1^), 50 (TCS1) and 100 µg·L^−1^ (TCS2) of TCS. Values are means ± SD (*n* = 30). a and b: Significant differences are indicated by different letters compared to control at *p* < 0.05 (ANOVA, post hoc, Tukey HSD test, STATISTICA 8.0).

TCS statistically reduced mussel’s filtration rate following concentration gradient. For mussels treated with TCS1, no statistical difference (*p* > 0.05) was observed between the treated and the untreated group. In contrast, the FR in mussels exposed to TCS2 was reduced from 115 ± 4.58 to 72 ± 4.08 mg·indiv^−1^·h^−1^ (Figure 1A) showing a statistical difference (*p* < 0.05) in comparison to the untreated group confirmed by the one-way ANOVA and Tukey’s HSD test (Figure 1A).

After 14 days of TCS treatment, respiration (RR) was also changed. A very low RR (from 0.69 ± 0.06 to 0.56 ± 0.011 mg·O_2_·h^−1^) was detected for mussels treated with TCS2 = 100 g·L^−1^, exhibiting a statistical difference (*p* = 0.0012), compared to untreated mussels. Respiration was reduced following the concentration of TCS (Figure 1B).

### 3.2. Effects of Triclosan on Oxidative Stress Biomarkers

**Figure 2:**
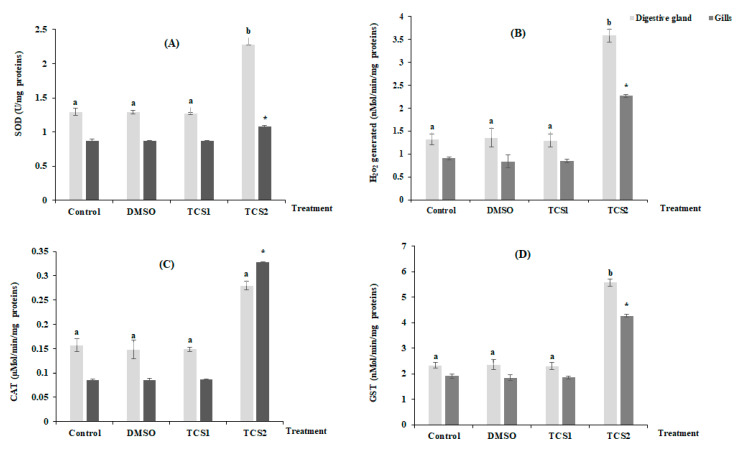
Activities of SOD (**A**), H_2_O_2_ level (**B**), CAT (**C**) and GST (**D**), in gills and digestive glands of untreated mussels and mussels treated with 50 and 100 µg·L^−1^ of TCS. Values are represented by means ± SD (*n* = 30). Significant differences in digestive glands are indicated by different letters (a and b) compared to control at *p* < 0.05. Asterisks indicate significant difference in the gills at *p* < 0.05 compared to relative controls (ANOVA, post hoc, Tukey HSD test, STATISTICA 8.0).

Figure 2 shows the mean variation of H_2_O_2_ production and antioxidant enzymes activities in considered organs after TCS contamination (Figure 2). No statistically, modifications for all considered parameters were found in the tissues of gills and digestive glands following TCS1 treatment. In contrast, treatment with TCS2 increased SOD activity by 25 and 76% in gills and digestive glands, respectively (Figure 2A). Additionally, mussels treated with TCS2 produced much more H_2_O_2_ in their gills and digestive glands. The digestive glands showed the biggest increase in H_2_O_2_ generation, with a mean value rising from 1.31 ± 0.11 nMol/min/mg proteins in the untreated group to 3.57 ± 0.14 nMol/min/mg proteins in the TCS2-treated group (Figure 2B). The exposure to TCS1 treatment was not followed by significant changes in CAT activity in either tissue (Figure 2C). However, the activity of this biomarker increased from 0.15 ± 0.013 µMol/min/mg proteins to 0.27 ± 0.009 µMol/min/mg proteins, in the digestive glands of mussels treated with TCS2 and from 0.08 ± 0.001 µMol/min/mg proteins to 0.32 ± 0.0009 µMol/min/mg proteins in the gills (Figure 2C).

GST showed a similar trend with an increase after exposure to TCS2, and a statistically significant change (*p* < 0.05) was recorded when compared activity of this marker between TCS2 treated mussels and control group (Figure 2D).

### 3.3. Effects of Triclosan on MDA Levels and AChE Activity

**Figure 3:**
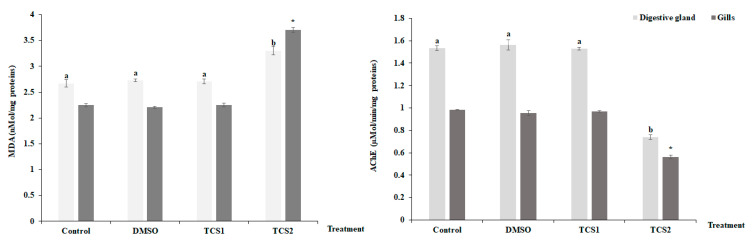
MDA level and Acetylcholinesterase (AChE) activity, in gills and digestive glands of untreated mussels and mussels treated with 50 and 100 μg·L^−1^ of TCS. Values are means ± SD (*n* = 30). a and b: Significant differences in digestive glands are indicated by different letters compared to control at *p* < 0.05. *, is significantly different in the gills at *p* < 0.05 compared to relative controls (ANOVA, post hoc, Tukey HSD test, STATISTICA 8.0).

After being exposed to TCS1, no appreciable alteration was seen for MDA contents in the mussels’ considered tissues (Figure 3). However, contents were found to be higher following TCS2 treatment.

TCS2 also altered AChE activity which dramatically decreased (Figure 3) in the digestive glands, decreasing from 1.53 ± 0.02 µMol/min/mg proteins to 0.73 ± 0.01 µMol/min/mg proteins, and in gills, decreasing from 0.98 ± 0.002 µmol·min^−1^·mg^−1^ proteins to 0.56 ± 0.015 µMol/min/mg proteins.

## 4. Discussion

Water pollution from drug residues is a growing environmental concern, according to water policy experts, and they advise the rigorous monitoring of certain of these substances to ascertain the damage they may cause to aquatic life [32].

The physiologic parameters measurements including filtration and respiration capacities on bivalve mollusks were used for chemicals ecotoxicity quantification [33,34]. They gave a hint as to the probable effects of environmental contaminants on aquatic population and subsequently on a marine ecosystem. In addition, these parameters gave an explanation to the short-term functional adjustments of mussel nutrition under perturbation. In the present study, filtration and respiration capacities were measured to evaluate the toxicity of pharmaceutical TCS in marine mussels *Mytilus galloprovincialis*. Filtration and respiration capacities results indicated that TCS reduces both parameters in mussels during two weeks of treatment with 100 µg·L^−1^ of this drug. As previously proposed for other organisms subjected to external stressors, this reduction may be connected to the bio-uptake of TCS in the mussel treated with high TCS concentration, which causes a decrease in oxygen intake and aerobic cellular energy generation [35]. Our findings are consistent with other research that found a decrease in the feeding rate following contamination and suggested that this response was caused by anxious animals striving to reduce their exposure to contamination [36]. No significant modification was observed for both filtration and respiration capacities after exposure to TCS1 = 50 µg·L^−1^. This could be due to the TCS concentration being lower in this study compared to other studies [37,38]. In addition, this result may probably confirm the resistance of mussels and its ability to avoid the contaminant at this level, as confirmed for other bivalve expose to pharmaceuticals [39]. According to these authors, mussels kept their valves closed to prevent the buildup of carbamazepine in order to deal with exposure to this chemical. Similar to this, Gosling [40] noted that bivalves have the ability to quickly seal their valves as a form of defense when confronted with a stressful situation. Thus, less energy could be needed for respiration and feeding as a result of this closure [41]. In contrast, a slowing in the mussels’ metabolic ability might be the cause of the decrease in filtration and respiration capacities shown at high TCS concentration. As documented by Oliveira et al. [39] for mussels subjected to different amounts of carbamazepine, this is leading to reduced energy consumption being employed in other biological mechanisms.

Crossing the physiological parameters, the TCS could be accumulated with the mussel’s tissues leading to biochemical changes. To keep away organisms from an overload of Reactive Oxygen Species (ROS) followed by an oxidative stress status, antioxidant defense enzymes are usually activated. These enzymes’ significant contribution to antioxidant defense in bivalves has been proven [42]. Antioxidant enzymes are induced when ROS are produced in response to pollutants on exposed bivalves. In the current investigation, mussels exposed to TCS2 level had significantly higher SOD and CAT activities in the target organs. Since these enzymes are also involved in O_2_^•−^ and H_2_O_2_ detoxification, this result supports the possibility of oxidative stress brought on by TCS exposure. Additionally, oxidative stress will develop if the generation of ROS rises [43]. In this work, the H_2_O_2_ content was increased in both organs after TCS2 treatment and this may potentially lead to antioxidant enzymes induction, especially CAT which acts as scavengers of hydrogen peroxide. A similar study has reported that SOD and CAT were induced in a concentration-dependent manner after exposure to diclofenac [44]. The authors demonstrated that Diclofenacs modulate SOD and CAT activities in stressed groups. The increase in SOD and CAT activities noticed in this study is a functional biological adjustment to neutralize oxidative stress related to ROS production after TCS exposure.

Phase II metabolism often involves the detoxification of TCS by the cytosolic multifunctional enzyme GST, which catalyzes the conjugation of several metabolites [45]. Additionally, earlier research revealed that the metabolism’s phase II (GST) was responsive to pharmaceutical concentrations [46]. In the current investigation, TCS2 treatment induced the GST activity in the considered organs.

Our results are in good agreement with a previous report [47] in which GST activity in *Perna perna* was only induced at the highest concentration of DCF. Previous investigations have also revealed an increase in this biomarker’s activity in aquatic invertebrates exposed to TCS [48,49].

TCS exposure modulates GST in a dose-dependent way, and researchers hypothesize that the formation of hazardous metabolites via lipo-peroxidation through the Fenton reaction is what causes the increase in GST. The optimization of oxidative stress related to ROS overproduction and the restoration of the cellular homeostasis may also be linked to the stimulation of GST activity [45]. The antioxidant enzyme responses of mussels to TCS exposure could lead to suggest that the impacts of pharmaceuticals are related to concentration and time as reported in previous investigations [50,51].

ROS overproduction could lead to cell membrane damage and MDA is a useful marker of oxidative damage when organisms are unable to handle oxidative stress [52]. In this work, the MDA level in mussels was increased following TCS2 treatment. According to similar findings, TCS exposure modifies MDA levels in zebrafish (*Danio rerio*), resulting in increased lipid peroxidation [53]. Furthermore, TCS increases ROS production, which damages the cell membrane, as explained by changes in MDA levels and oxidative biomarkers [52]. This disruption may be related to lipid synthesis and a degradation in treated mussels [54]. Because lipid peroxidation is sign of cell function loss under oxidative stress, the MDA response could explain the toxic effect of TCS [55]. Aside from antioxidant biomarkers, AChE can be used to predict significant or permanent long-term damage [46]. TCS2 treatment also reduced AChE activity in mussels’ organs, indicating that this compound has a toxic effect in marine mussels. Oliveira et al. [56] observed this inhibition in TCS-exposed zebrafish.

The obtained results in this study indicate that both physiological and biochemical analysis tests are more sensitive than the usual toxicity test (lethal concentration) and constitute a useful tool to measure the impact of TCS in marine mussels.

## 5. Conclusions

The mussel *M. galloprovincialis* exposed to TCS was affected during 14 days of the study. The physiological response and the antioxidant system were rapidly triggered in the gills and digestive glands. Evidence of cell membrane damage was also observed. Furthermore, the TCS induced AChE activity in a concentration-dependent manner and both organs were affected, suggesting a possible breakdown of the cholinergic neurotransmission functions. This investigation highlighted the important association of physiological impairments and biochemical changes in pharmaceutical toxicity determination. Furthermore, based on ecotoxicological data, it is also important to examine the possible degradation of TCS in marine ecosystems to reduce its concentration and then protect the environment.

## Data Availability

All the data in the article are available from the corresponding author upon reasonable request.
